# Dry Formulation of Virus-Like Particles in Electrospun Nanofibers

**DOI:** 10.3390/vaccines9030213

**Published:** 2021-03-03

**Authors:** Sasheen Dowlath, Katrin Campbell, Farah Al-Barwani, Vivienne L. Young, Sarah L. Young, Greg F. Walker, Vernon K. Ward

**Affiliations:** 1 Department of Microbiology & Immunology, School of Biomedical Sciences, University of Otago, P.O. Box 56, 720 Cumberland St, Dunedin 9054, New Zealand; sasheen.dowlath@yahoo.co.nz (S.D.); farahalbarwani@gmail.com (F.A.-B.); vivienne.young@otago.ac.nz (V.L.Y.); 2 Department of Pathology, Dunedin School of Medicine, University of Otago, P.O. Box 56, 720 Cumberland St, Dunedin 9054, New Zealand; katrin.campbell@otago.ac.nz (K.C.); sarah.young@otago.ac.nz (S.L.Y.); 3 Faculty of Medicine and Health, School of Medical Sciences, The University of Sydney, Sydney, NSW 2006, Australia; 4 School of Pharmacy, University of Otago, P.O. Box 56, 720 Cumberland St, Dunedin 9054, New Zealand; greg.walker@otago.ac.nz

**Keywords:** electrospinning, nanofiber, dry-formulation, virus-like particle, vaccine

## Abstract

Biologics can be combined with liquid polymer materials and electrospun to produce a dry nanofibrous scaffold. Unlike spray-drying and freeze-drying, electrospinning minimizes the physiological stress on sensitive materials, and nanofiber mat properties such as hydrophobicity, solubility, and melting temperature can be tuned based on the polymer composition. In this study, we explored the dry formulation of a virus-like particle (VLP) vaccine by electrospinning VLP derived from rabbit hemorrhagic disease virus modified to carry the MHC-I gp100 tumor-associated antigen epitope. VLP were added to a polyvinylpyrrolidone (PVP) solution (15% *w*/*v*) followed by electrospinning at 24 kV. Formation of a nanofibrous mat was confirmed by scanning electron microscopy, and the presence of VLP was confirmed by transmission electron microscopy and Western blot. VLP from the nanofibers induced T-cell activation and interferon- (IFN-) γ production in vitro. To confirm in vivo cytotoxicity, Pmel mice treated by injection with gp100 VLP from nanofibers induced a gp100 specific immune response, lysing approximately 65% of gp100-pulsed target cells, comparable to mice vaccinated with gp100 VLP in PBS. VLP from nanofibers also induced an antibody response. This work shows that electrospinning can be used to dry-formulate VLP, preserving both humoral and cell-mediated immunity.

## 1. Introduction

Virus-like particles (VLP) have been extensively explored as vaccine material, with current VLP vaccines comprising liquid suspensions. VLP are structures derived from the shell components of a wild-type virus and are therefore classed as subunit vaccines. VLP are devoid of a viral genome and cannot infect or replicate within the host [1,2]. Due to the antigenic and structural similarities, VLP have the ability to induce immunity against the parental virus and are currently utilized in this capacity in licensed vaccines against human papillomavirus (HPV) [3], hepatitis B virus (HBV) [4], and hepatitis E virus (HEV) [5,6]. VLP can also be modified to carry unrelated antigens such as components of other pathogens or cancer epitopes for therapeutic vaccines, thus making them a versatile and safe particulate vaccine delivery platform [7,8,9,10].

Like many vaccines, liquid formulations of VLP vaccines must be correctly stored and strictly maintained between 2–8 °C to preserve vaccine potency, adding both cost and complexity to the supply chain [11,12,13]. A broken cold chain could result in wasted vaccine stock or the administration of ineffective vaccines. This problem is often compounded in countries where cold chain infrastructures may be suboptimal [14]. Vaccine suspensions can include a variety of compounds to improve stability including salts, pH buffers, cryoprotectants, and thermoprotectants. [13]. Despite advancement in formulation, refrigeration, and storage technology, inadequate temperature control remains the primary cause of compromised vaccine stocks [11,12,14]. One possible approach to minimize the reliance on cold-chain handling is to prepare a dry formulation of the vaccine material, which can be reconstituted into a liquid suspension prior to administration. Dry formulations involve the removal of water from the sample, reducing the motility of the sample and minimizing degradation pathways facilitated by water. [15]. Dry formulations are less prone to temperature-induced degradation, leading to reduced reliance on the cold chain, fewer wasted vaccine stocks, and an increase in cost-effectiveness and shelf life [16]. Another key advantage is that dry formulated material can be packaged into capsules, nasal sprays, inhalers, and transdermal patches, enabling flexibility with administration routes and target sites [16].

Freeze-drying has historically been a gold standard for dry formulation of biologics and pharmaceuticals; however, freeze-drying was shown to result in undesirable aggregation and attenuated immunogenicity of VLP [17]. Freeze-drying is also a long, costly, and energy consuming process, which requires expensive equipment [15]. Some success has been garnered by freeze-drying the leaves of a plant-based expression system containing the VLP, which may be used in oral vaccination applications such as capsules [18]. Spray-drying is another technology which has showed promise. A liquid suspension containing vaccine material and excipients are nebulized into an aerosol, before being sprayed into a heated gaseous medium for dehydration into a powder [16]. Sheer stress generated during the nebulization process may result in the damage of protein-based vaccine components, while the dehydration stress may also cause undesirable aggregation and attenuation [19,20,21]. Saboo et al. reported successful spray drying of HPV VLPs into a dry powder, although this was only possible using a multi-component excipient system due to stresses exhibited during the process [22]. The research paradigm is shifting towards eliminating the cold chain altogether, with innovations in vaccine formulations and storage technology whereby the vaccine material is maintained to the highest integrity with exposure to the least amount of stress [23,24,25].

Electrospinning is a one-step technique that involves the rapid drying of a polymer solution into a fibrous sheet by applying an electrostatic field to the solution [26]. The electrostatic field facilitates the formation of a charged nanofiber jet, which is attracted to a grounded surface. During the time of flight, the solvent rapidly evaporates, leaving behind a continuous solid nanofiber filament, which is then randomly deposited on the grounded collection surface and over time, a nanofiber sheet is formed [27]. Electrospinning offers several advantages compared to conventional dry formulation methods such as freeze-drying or spray drying. The solvent is removed rapidly from the liquid formulation, typically in nanoseconds, as opposed to several days with freeze-drying [15,25,28]. Temperature and humidity control is often recommended but not essential and electrospinning can be carried out at ambient temperature and atmospheric pressure. The formulation is not subjected to extreme temperatures or pressure changes associated with freeze-drying and spray drying, allowing for a gentler drying process for sensitive biologics and proteins [16]. Nanofibers can be comprised of singular polymers, or mixture of polymers depending on the application. Properties such as high surface area to volume ratio, solubility, hydrophobicity, individual fiber size, and rate of dissolution can be altered by adjusting the polymer mixture [29]. Nanofiber technology has been employed in controlled release drug delivery, tissue regeneration, air and water filtration, and wound healing applications [26,29,30,31,32,33]. Scalability has also been improved in recent years with advancements in multi-needle and needle-less systems.

PVP was selected as the polymer for our nanofiber formulation due to its high-water solubility and is commonly used as a binding agent and drug stabilizer in oral tablets and capsules. PVP has previously been used as the basis for many electrospinning studies, including wound healing, the stabilization of drugs and bacteriophages, and other slow-release applications [34,35,36,37,38,39,40].

This study aimed to dry-formulate an active vaccine component in a nanofiber mat while maintaining immunogenicity. To demonstrate this, we used rabbit hemorrhagic disease virus (RHDV) VLPs as an antigen carrier that was modified to express an MHC-class I-restricted tumor antigen. The VP60 gene of RHDV was modified to fuse two copies of the human gp100 MHC-I epitope to the N-terminus, placing copies of the epitope internally in the assembled VLP [8]. The gp100 antigen was shown to elicit a strong cell-mediated immune response in tumor trials and was established as a murine melanoma model [8,41,42,43]. Previous studies also demonstrated that RHDV VLPs carrying heterologous antigens as therapeutic cancer vaccines, such as gp100, were able to induce a strong, enhanced immune response [8,41,44,45].

In this study, we successfully electrospun VLP into a PVP-based nanofiber and demonstrated that intact particles can be recovered and visualized by transmission electron microscopy (TEM) upon nanofiber solubilization. Furthermore, we showed that the humoral and cell-mediated immune responses were conserved when particles were stored in a nanofiber. Importantly, we demonstrated that both the cellular and humoral immune response generated by the dry-formulated VLP was comparable to freshly prepared VLP, both in vitro and in vivo.

## 2. Materials and Methods

### 2.1. Expression and Purification of VLP

**Figure 1:**
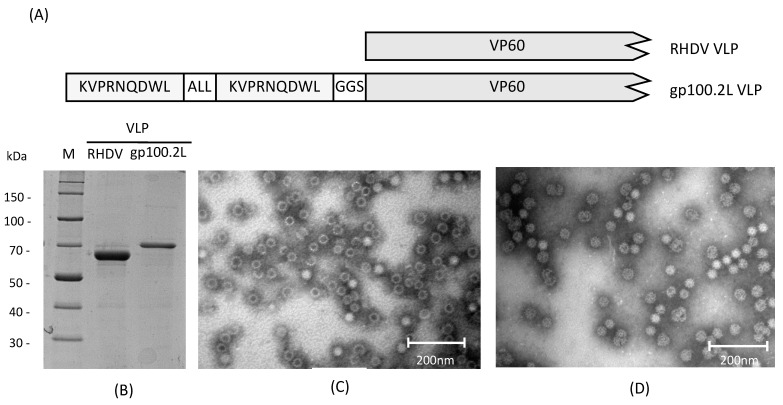
Expression and purification of virus-like particle (VLP). (**A**) Gene constructs used for expression of unmodified rabbit hemorrhagic disease virus (RHDV) VLP and RHDV VLP containing two gp100 epitopes (gp100.2L VLP). The 60 kDa capsid protein of RHDV is represented as VP60. Two copies of the gp100 epitope KVPRNQDWL are shown in the gp100.2L construct, along with a processing linker (ALL) and a flexible linker (GGS) at the junction between the gp100 epitopes and the VP60 sequence. (**B**) Ten percent SDS-PAGE of purified RHDV VLP and gp100.2L VLP stained with Coomassie Brilliant Blue G-250. (**C**,**D**) Transmission electron microscopy confirming particle assembly for RHDV and gp100.2L VLP, respectively. Upon overexpression, 180 copies of the VP60 monomer self-assemble into icosahedral capsids of approximately 40 nm in size.

VLP were expressed in *Sf*21 insect cells using a baculovirus expression system as previously described [46,47]. DNA encoding two copies of the gp100 epitope (KVPRNQDWLALLKVPRNQDWL) with an immunoproteasome cleavage sequence (ALL) was inserted at the N-terminus of the RHDV VP60 gene (Figure 1A) by PCR extension and used to generate a recombinant baculovirus by homologous recombination (gp100.2L VLP). Expression and purification of VLP were carried out as previously described [47]. Briefly, suspension cultures of Sf21 insect cells were infected with recombinant baculovirus expressing the VP60 protein +/− the gp100 epitopes at a multiplicity of infection of 1 and incubated at 27 °C at 125 rpm for 3 days. The cells were lysed with 0.5% Triton X-100 and the VLP purified using differential centrifugation, followed by a centrifugation on a 1.2 and 1.4 g mL CsCl step gradient at 100,000 g for 18 h. The VLP band was harvested and dialyzed into 0.1 M phosphate buffer pH 7.4 containing 0.15 M NaCl (PBS). A control VLP without the gp100 epitope motif was also expressed (RHDV VLP). Expression and purification of VLP was confirmed by SDS-PAGE and the presence of the gp100 epitope was confirmed by mass spectrometry at the Otago Centre for Protein Research. Particle assembly was confirmed by TEM at the Otago Centre for Electron Microscopy. 

### 2.2. Electrospinning

Polyvinylpyrrolidone (PVP K-90, Mw 360,000, Sigma-Aldrich, St. Louis, MO, USA) solutions were prepared in Milli-Q water with stirring overnight at room temperature. VLP (5 mg) which had been dialyzed against PBS was added to a 30% *w*/*v* PVP solution at a 1:1 ratio and mixed by magnet stirring for 20 min. The solution was drawn into a 5-mL syringe (Becton Dickinson, Franklin, NJ, USA) with blunted needle (18 G) and attached to an infusion pump (Fusion Touch 100, Chemyx Inc., Stafford, TX, USA) to deliver the solution at a constant flow rate of 0.1 mL h^−1^. The live terminal of a high voltage power supply was attached to the needle and was set to generate an electrical potential of 24 kV. A grounded metal wheel collector was placed 15 cm from the needle tip and rotated at approximately 10 rpm. A strip of aluminum foil was placed around the circumference of the wheel, to which nanofiber was deposited. Nanofiber mats were stored at room temperature on the aluminum foil in a zip-locked bag containing silica gel. Nanofibers were stored for up to seven days prior to biological assays. 

### 2.3. Electron Microscopy

Carbon-coated TEM grids were plasma discharged and 10 µL of purified VLP (0.2 mg ml ^−1^) was deposited onto the grid for 1 min at room temperature. The grid was stained with 10 µL of 1% phosphotungstic acid (PTA) pH 6.8 and imaged using a Philips CM100 TEM electron microscope. Nanofiber mat samples (1 cm^2^) were fixed to SEM sample stubs before being coated with 5 nmol gold-palladium using an Emitech K575X Peltier-cooled high-resolution sputter coater fitted with a 250X carbon coater and examined by SEM using a variable-pressure scanning electron microscope (Carl Zeiss Inc., Oberkochen, Germany). 

### 2.4. Western Blot

A 0.1 cm^2^ section of the nanofiber mat was dissolved and heated at 100 °C for 5 min in SDS-PAGE sample buffer before being loaded onto a 10% SDS-PAGE gel alongside VLP standards (0.0625–4 µg). The resolved proteins were transferred onto nitrocellulose membrane (Amersham protran, GE Life Sciences, Chicago, IL, USA) then blocked with 0.1% *w*/*v* casein alanate in Dulbecco’s PBS (DPBS). The membrane was probed with anti-VP60 rabbit polyclonal antibody diluted 1:1000 in PBS + 0.1% *w*/*v* casein alanate and the membrane washed three times with PBS +0.1% *v*/*v* Tween-20. The primary antibody was detected using a 1:10,000 dilution of donkey anti-rabbit IgG antibody (SA5-10044, ThermoFisher Scientific, Waltham, MA, USA) in PBS + 0.1% casein alanate and the membrane washed prior to detection. Band intensity was imaged using a LI-COR Odyssey FC (800 nm) and analyzed on Image Studio. Fluorescence values for the standard curve were plotted to fit a linear regression model, which was used to quantify the nanofiber protein concentration. 

### 2.5. Animals and Ethics

Female C57BL/6 mice were obtained from the Hercus Taieri Research Unit, University of Otago, Dunedin, New Zealand. Female pmel mice, expressing T-cell receptors specific to the MHC-I restricted gp100_25–33_ epitope, were obtained from the Young lab breeding stock, Department of Pathology, University of Otago, Dunedin New Zealand. Experiments were conducted in accordance with an ethical permit granted by the University of Otago Animal Ethics Committee (AEC D103/17). Animals were euthanized by carbon dioxide or cardiac puncture.

### 2.6. T-Cell Proliferation and Interferon-γ Production Assay

Bone marrow-derived dendritic cells (BMDCs) were extracted from femurs and tibiae of C57BL/6 mice as previously described [48], adjusted to a concentration of 5 × 10^4^ cells ml^−1^ and pulsed with either PBS, PVP, blank nanofiber, 1 µg RHDV VLP or gp100.2L VLP, or gp100_25–33_ peptide (0.38 μg·mL^−1^, the molar equivalent of 1 µg of gp100.2L VLP). A section of nanofibers containing 1 µg of RHDV VLP or gp100.2L VLP was added directly onto the cells. T-cells from pmel mice were prepared according to Kramer et al. [41] and sorted for CD8 expression using anti-CD8 AutoMACS MicroBeads (Miltenyi Biotech, Bergisch Gladbach, Germany). CD8+ T cells (1 × 10^8^ cells·mL^−1^) were diluted 1:1 with 20 µM carboxyfluorescein succinimidyl ester (CFSE) (Invitrogen) and added to the pulsed BMDCs at a ratio of 10:1, and then incubated for 72 h at 37 °C, 10% CO_2_. Cells were treated with 50 µL of a 1:1000 dilution of LIVE/DEAD™ Fixable Near-IR Dead Cell Stain (Life Technologies™, Eugene, OR, USA) followed by treatment with CD16/CD32 Fc blocking antibody, and then stained with CD3-PE-CF594 (clone 145-2C11) and CD8α-APC (clone 53-6.7) (Life Technologies™, Eugene, OR, USA). Fluorescence was measured using a Gallios flow cytometer (Beckman Coulter, Brea, CA, USA) with a three-laser (405, 488, and 633 nm), ten-color configuration and analyzed using Kaluza software (Beckman Coulter, Brea, CA, USA). Interferon-γ (IFN-γ) levels were measured by enzyme-linked immunosorbent assay (ELISA) as previously described [41]. 

### 2.7. In Vivo Cytotoxicity

Five groups of six mice were vaccinated with either PBS, 100 µg of gp100.2L VLP, 100 µg of RHDV VLP from a dissolved nanofiber sample, 100 µg equivalent of gp100.2L VLP from a dissolved nanofiber sample, and the molar equivalent of free gp100 contained in 100 µg of VLP (100 µL at 19.6 µg·mL^−1^). Each vaccination contained 25 µg of CpG oligonucleotide as an adjuvant (CpG 1826, GeneWorks, SA, Australia) added to the treatment prior to vaccination. For nanofiber samples, this was post-dissolution in PBS. Treatments were administered subcutaneously to the left flank using a 29-G needle. Mice were boosted 21 days later with an additional dose of their respective treatment. Mice were injected intravenously with target (pulsed with gp100 peptide) and non-target splenocytes on day 28 derived from donor C57BL/6 mice. The red blood cells were removed from the splenocyte preparation by lysis with ammonium chloride followed by centrifugation. Remaining cells were divided into 2 groups; unpulsed (untreated) cells and pulsed cells, treated with 10 mM of synthetic gp100_25–33_ peptide (KVPRNQDWL) and incubated for 2 h at 37 °C, 5% CO_2_. Each group was differentially stained, with the untreated group receiving 5 µM CFSE (CFSE^LO^) and the treated group receiving 50 µM CFSE (CFSE^HI^). Cells from each group were mixed in a 1:1 ratio and 1 × 10^8^ cells were injected into the tail vein of each mouse. Mice were sacrificed 40 h post injection and the splenocytes were harvested and stained with LIVE/DEAD™ Fixable Near-IR Dead Cell Stain. Fluorescence was measured on a Gallios flow cytometer and analyzed using Kaluza software. 

### 2.8. Serum Collection and ELISA

Mice from the in vivo cytotoxicity assay were bled on day 30 by cardiac puncture, serum was collected and stored at −80 °C. Each well of a 96-well plate was coated with 2 µg of VP60 followed by incubation at 4 °C overnight and then washed in PBS + 0.05% Tween-20 six times (wash buffer). The plate was blocked by adding 100 µL of 20 mg mL^−1^ BSA in PBS (blocking buffer) per well and incubated for an hour at 37 °C and then washed six times. Serum was serially diluted ten-fold (10^−1^–10^−6^) in blocking buffer and 100 µl added to each well. Plates were incubated for 2 h at 37 °C and then washed six times in wash buffer. Anti-mouse IgG-HRP (Sigma-Aldrich, Cat. no. A9044) was diluted 1:60,000 in blocking buffer before addition of 100 µl to each well and incubated for 1 h at 37 °C. Wells were washed six times before addition of 100 µL 3,3′,5,5′-tetramethylbenzidine (TMB) substrate to each well. The reaction was stopped after 2 min by adding 100 µL of 1 M HCl solution. Plates were read at 450 nm using a Multiskan FC Microplate spectrophotometer (ThermoFisher Scientific).

### 2.9. Statistical Analysis

Statistical analysis was carried out using GraphPad Prism version 7.04 (GraphPad Software, La Jolla, CA, USA). Analyses of the in vivo data consisted of a Kruskal Wallis non-parametric test with a Dunn’s post-hoc test. Advice on statistical analysis was provided by Andrew Gray, Biostatistical Unit, Division of Health Sciences, University of Otago. 

## 3. Results

### 3.1. VLP Expression and Purification

VLP were expressed using the commercial baculovirus expression system, flashBAC Ultra (Oxford Expression Technologies, Oxford, UK). VLP were purified by differential centrifugation and a CsCl gradient before assessment by SDS-PAGE (Figure 1B). Protein identity was confirmed by Western blot and the presence of the correct gp100 sequence was confirmed by mass spectrometry analysis (data not shown). Overexpression of the VP60 capsid protein led to self-assembly into icosahedral particles of approximately 40 nm in size, which was confirmed by TEM (Figure 1C,D, respectively). Size and shape of the expressed VLP were consistent with previous work [46].

### 3.2. Incorporation of VLP into Nanofibers

**Figure 2:**
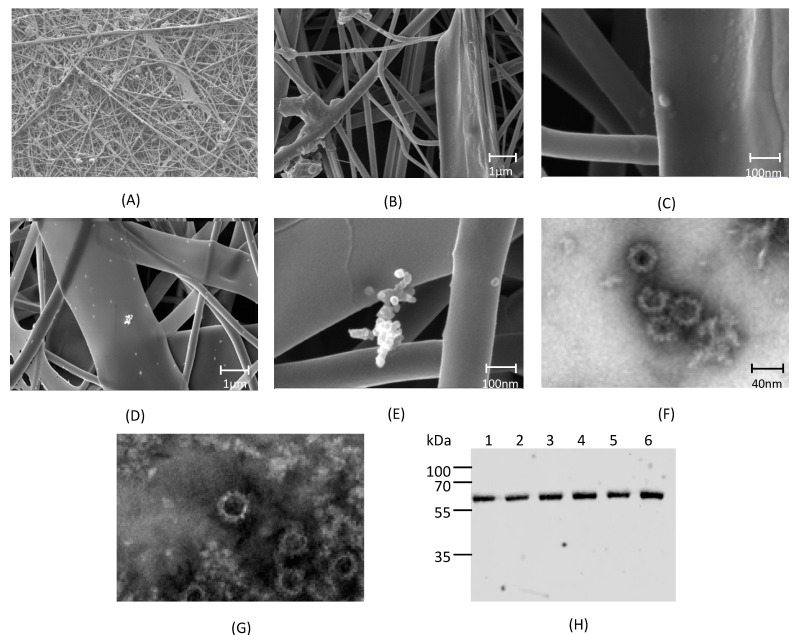
Incorporation and analysis of VLP in electrospun nanofibers. (**A**) Macrostructure of a nanofiber mat produced by electrospinning a 15% *w*/*v* polyvinylpyrrolidone (PVP) solution. (**B**,**C**) SEM of blank nanofiber microstructures. (**D**,**E**) SEM of RHDV VLP-loaded nanofiber microstructures. (**F**,**G**) RHDV-loaded nanofibers were dissolved and re-purified on a CsCl gradient, recovered material viewed by TEM. (**H**) Nanofiber loading uniformity. Lanes 1–6 represent randomly selected samples cut from the nanofiber mat and dissolved. An amount equivalent of 0.1 cm^2^ nanofiber was run in each lane and VP60 levels compared by fluorescent western blot using an anti-RHDV VP60 antibody. The original western blot and relatively band intensities are shown in Supplementary Figure S1.

Electrospinning of VLP in a 15% (*w*/*v*) PVP solution in PBS was performed successfully at 24 kV and a flow rate of 0.1 mL h^−1^. While PVP-water solutions can be electrospun at much higher flow rates, PBS was a necessary addition to the formulation in order to preserve the structural integrity of the VLP. SEM imaging (Figure 2A) of an electrospun nanofiber mat showed heterogenous nanofiber structures including tubular strands and flat ribbon-like sheets with diameters ranging from 100 nm to 5 µm. A comparison between the blank, unloaded nanofibers (Figure 2B,C) and the RHDV VLP-loaded nanofibers (Figure 2D,E) revealed particles on the surface of the VLP-loaded nanofiber, consistent with the presence of RHDV VLP. The observed particles were arranged singularly or clustered and the diameter of the particles was consistent with sputter-coated VLP. To confirm the identity of the observed structures, a VLP-loaded nanofiber mat was dissolved in PBS and then loaded onto a 1.2 and 1.4 g·cm^−3^ CsCl step gradient as utilized for VLP purification. Material recovered from the CsCl interface revealed that intact VLP particles were present, as visualized by TEM (Figure 2F,G). SDS-PAGE confirmed the protein to be 60 kDa in size, consistent with the size of VP60. To confirm that the isolated particles were of RHDV VP60 origin and simultaneously assess nanofiber loading uniformity, 0.1 cm^2^ samples were dissolved and analyzed by Western blotting (Figure 2H). Results confirm that the 60 kDa protein was VP60 and that loading was relatively consistent across the mat. These results indicate that VLP can be electrospun into a dry nanofibrous mat and that intact VLP can be recovered upon dissolution of the mat.

### 3.3. T-Cell Proliferation and IFN-γ Production Assays

**Figure 3:**
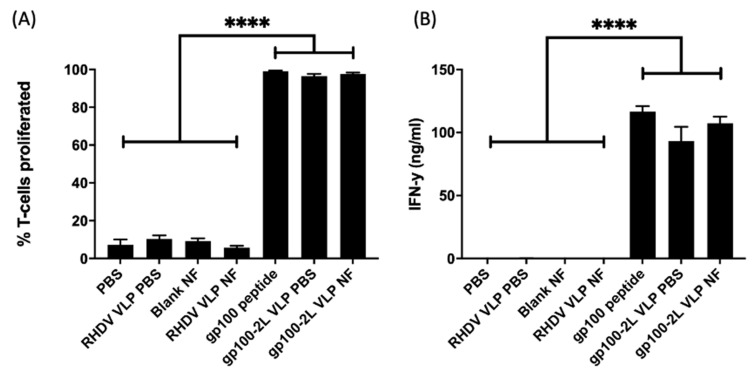
In vitro T-cell proliferation and cytokine response towards VLP-loaded nanofibers. BMDCs Cells were pulsed with 1 µg of VLP in PBS, 1 µg of VLP from a nanofiber, or gp100 peptide at a molar equivalent to gp100 in 1 µg of VLP. T-cells were co-cultured with pulsed bone marrow-derived dendritic cells (BMDCs) for 72 h. (**A**) T cell proliferation determined by flow cytometry and (**B**) IFN-γ production results determined by ELISA of the supernatant. Each bar represents the mean percentage of T-cell proliferation (±SEM). The data represents 3 independent experiments. Statistical significance was determined by a one-way ANOVA with Dunnett’s multiple comparisons test. **** *p* < 0 0001. Statistical significance displayed is the comparison of RHDV VLP nanofiber against negative control groups. The gating strategy used to generate this data is shown in Supplementary Figure S2.

An initial in vitro test was performed to confirm activity before analysis in vivo. The VLP-loaded nanofibers were tested for the ability to induce a cell-mediated immune response by measuring T-cell proliferation as well as IFN-γ production. BMDCs from C57BL/6 mice were pulsed with treatments, followed by a co-culture with T cells from pmel mice, which were genotyped to express a T-cell receptor specific to the gp100_25–33_ peptide. RHDV VLP-loaded nanofiber (negative control) and gp100.2L VLP-loaded nanofiber samples were placed directly into the cell culture. Other controls included VLP (no epitope) in PBS, gp100.2L VLP in PBS, and gp100 peptide as a positive control. T-cell proliferation data (Figure 3A) was standardized against the gp100 peptide response. The gp100.2L VLP-loaded nanofiber induced a very strong response at or near 100%, similar to other groups containing gp100. In contrast, RHDV VLP-loaded nanofiber without the gp100 epitope induced limited proliferation. Similarly, gp100.2L VLP-loaded nanofiber induced an IFN-γ response of 111 ng ml^−1^ (Figure 3B), comparable to that of the gp100 peptide (116 ng mL^−1^). 

### 3.4. In Vivo Cytotoxicity and Antibody Response

**Figure 4:**
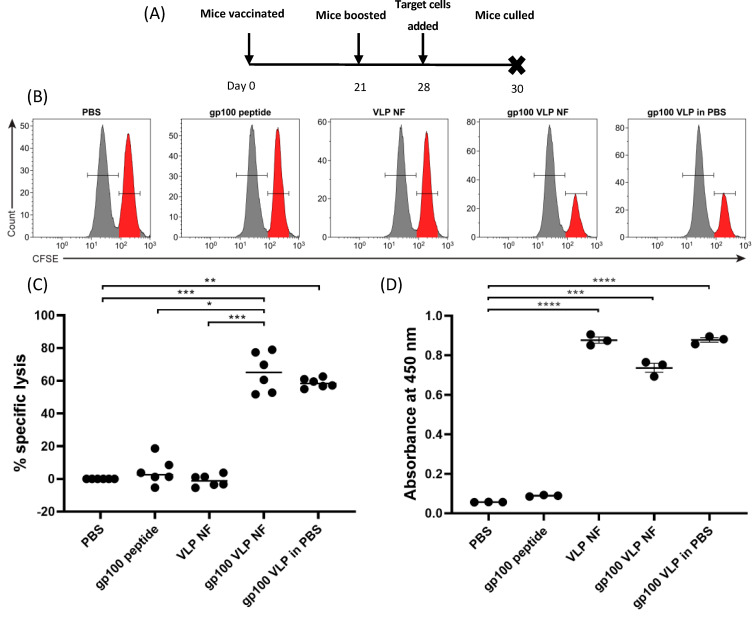
In Vivo cytotoxicity and antibody production. (**A**) Cytotoxicity assay timeline. *n* = 6 mice per group were vaccinated on day 0 with a PBS control, 100 µg RHDV VLP in a PVP nanofiber, 100 µg gp100.2L VLP in PBS, 100 µg gp100.2L VLP in a PVP nanofiber, or gp100 peptide at a molar equivalent to that on 100 µg VLP. All treatments were supplemented with 25 µg CpG. On day 21, mice were boosted with the same treatments, followed by injection with fluorescently labelled target cells on day 28. Mice were sacrificed 40 h later, and target cells analyzed by flow cytometry for specific lysis. (**B**) Representative histograms depicting the relative proportions of non-target (unpulsed CFSE^LO^; grey gate, left) and target (gp100_25–33_ peptide-pulsed CFSE^HI^; red gate, right) cells isolated from various treatment groups after 40 h. (**C**) Specific lysis of target cells administered intravenously. Data points are percent of specific lysis for single mice compared to the average control group (PBS) percent of specific lysis. Statistical significance was determined by a Kruskal–Wallis test with Dunn’s multiple comparisons test. *** *p* < 0.001, ** *p* < 0.01, * *p* < 0.05. The gating strategy used to generate this data is shown in Supplementary Figure S3. (**D**) Antibody ELISA. Serum from *n* = 3 mice was diluted 1:10,000 and screened for antibody production against VP60 by ELISA. Assays were performed three times in triplicate for each animal. Data points represent the average for each animal from three assays. The bar represents the mean and SEM across all samples. Statistical significance was determined by one-way ANOVA with Tukey’s multiple comparisons test. **** *p* < 0.0001, *** *p* < 0.001, ** *p* < 0.01, * *p* < 0.05.

Following demonstration that T-cell activation and IFN-γ production was upregulated by VLP released from nanofibers, the activity of VLP-loaded nanofiber was assessed in vivo for the induction of target specific cytotoxicity towards gp100, as well as a specific antibody response toward the VLP. Mice were vaccinated subcutaneously with treatments followed by a boosting vaccination 21 days later (Figure 4A). On day 28, mice were challenged with unpulsed control splenocytes and gp100-pulsed target splenocytes. Mice were culled 40 h later and assessed for specific killing of the target cells by flow cytometry. Results from Figure 4B,C show that nanofibers loaded with gp100.2L VLP induced specific lysis of gp100-pulsed BMDCs (approximately 65%) in a similar capacity to that of gp100.2L VLP delivered in PBS (approximately 60%). While the results for the nanofiber gp100 VLP treatment produced a broader range of response than gp100 VLP without nanofiber, there was no statistical difference between these two VLP treatment groups carrying the gp100 epitope. PBS only and RHDV VLP-loaded nanofiber containing no epitope induced no specific lysis (Figure 4B,C). To assess ability of the released VLP to stimulate an antibody response, an enzyme-linked immunosorbent assay (ELISA) was performed using the collected serum as the source of the primary probing antibody against purified VLP (Figure 4D). Results indicate that all treatments that contained VLP induced an antibody response. RHDV VLP nanofiber and gp100.2L VLP nanofiber producing comparable antibody titers to that of the gp100.2L VLP delivered in PBS.

## 4. Discussion

Thermally stable vaccines could revolutionize and improve the efficiency and efficacy of vaccination regimes by greatly reducing costs, reducing vaccine wastage, allowing for an extended shelf-life, and minimizing the administration of ineffective vaccines. Electrospinning offers rapid drying of a polymer solution at room temperature, coupled with a cheaper running cost and a faster material preparation time when compared to other vaccine drying technologies, such as freeze-drying or spray drying [16]. In this study, we have demonstrated that electrospinning is an effective method to produce a dry formulation of a particulate vaccine. More importantly, results of the in vivo experiments demonstrate that VLP formulated in the nanofibers were able to stimulate a cell-mediated and humoral immune response without loss of functionality when compared to VLP that had not been incorporated into nanofibers. 

VLP were successfully electrospun in a PVP solution into a nanofiber mat. Inspection by SEM confirmed that VLP-like structures were visible on the surface of the nanofibers, indicating a successful drying of VLP. Western blot analysis of the dissolved gp100.2L VLP nanofiber confirmed that the material was of RHDV VP60 origin and that the material was evenly distributed throughout the sheet. Repurification of a dissolved nanofiber on a CsCl gradient followed by TEM analysis confirmed that intact particles were present and that structures were intact. These results indicated that VLP can be easily incorporated and recovered from a nanofiber while remaining structurally intact. For the nanofiber to be an effective dry formulation of a vaccine, it is of utmost importance to also maintain immunogenicity. 

As a proof of concept and further evidence to suggest the VLP was intact and able to elicit an immune response, VLP-loaded nanofibers were subjected to both in vitro and in vivo immune assays. The gp100 epitope was previously shown to establish a strong immunotherapeutic response in murine melanoma models, which can be further enhanced by delivery on a modified VLP, thus, providing a means of evaluating the immunogenicity of the dry formulated VLP [41]. Results in vitro indicated that the gp100 peptide was effectively cleaved and processed from the VLP particles by DCs, followed by cross-presentation in the context of MHC-I in order to stimulate a T cell proliferation and IFN-γ response. Results of the in vivo cytotoxicity assay demonstrated a significant response in the group vaccinated with the gp100.2L VLP nanofiber, similar to that of mice vaccinated with gp100.2L VLP delivered in PBS. Contrastingly, the gp100_25–33_ free peptide showed very little cytotoxic stimulation. It is important to note that longer, more complex peptides presented on a VLP require uptake and endosomal processing by antigen presenting cells, leading to more efficient cross-presentation on MHC-I when compared to shorter, free peptides such as gp100_25–33_ alone [8,41,49]. The comparable nature of the cytotoxic responses seen in the gp100.2L VLP nanofiber and gp100.2L VLP in PBS groups further suggest that the nanofiber VLP are intact and are processed by cross-presentation, as has been shown for free VLP [50]. 

Serum from the mice in the in vivo cytotoxicity work was harvested and tested for antibody production against VP60. All groups containing VLP, including in nanofibers, showed an upregulated antibody titer when compared to PBS and gp100 peptide groups. The similar antibody responses to VLP derived from the electrospun nanofiber and VLP that was not electrospun confirms that VP60 remains immunologically relevant in the nanofiber.

This work demonstrates an early proof of concept to show that VLP can be electrospun into a dry formulation nanofiber, and can be reconstituted prior to delivery while maintaining immunogenicity. The tuneability of the nanofiber enables the physical and chemical properties to be altered by adjusting the polymer composition. Due to the high water-solubility of PVP, reconstitution involved immersion in an aqueous solution, PBS in this case. Polymer choices or blends other than the PVP used in this study could be explored for fine tuning solubility, permeability, hydrophobicity and humidity stability, or to offer slow-release characteristics to enhance the immune response through the use of non-aqueous polymers. Other stabilizing materials such as trehalose can also be incorporated into the polymer solution [40]. Nanofibers allow for transportation and storage in the dry form up until the need for administration, or can be compressed into alternative delivery forms, such as oral capsules or patches which can be placed directly onto mucosal sites. Several doses could also be electrospun into a single, large, multidose sheet. A nanofiber formulation has the potential to also be delivered as a dry patch, to be hydrated by the tissue. 

Several factors, such as polymer concentration, electrostatic field strength, fluid viscosity, surfactants, distance to the collection surface, ionic strength, and flow rate can impact on the efficiency and quality of the final nanofiber [51,52]. Extending the research into the effects of electrospinning parameters on our PVP blend could improve electrospinning efficiency and protein yield.

## 5. Conclusions

This work demonstrates that electrospinning offers an attractive method for the dry formulation of particulate vaccines with complex biological structures without loss of functionality. VLP were successfully electospun into a nanofibrous mat and recovered upon reconstitution of the mat. By using VLP as an antigenic carrier stored in the nanofibers, we were able to confirm that both the humoral and cell-mediated immune response was maintained when VLP were released from the nanofiber mat and used as a vaccine. It should be noted that VLP was used as an exemplar antigen, and that this technique has the potential to be applied to a range of vaccine types and antigens.

## Data Availability

The data presented in this study are available in the manuscript and associated supplementary materials.

## Supplementary Materials

The following are available online at https://www.mdpi.com/2076-393X/9/3/213/s1, Figure S1: Nanofibre loading uniformity. Lanes 1–7 represent randomly selected samples cut from the nanofibre mat. Lane 1, 0.05 cm^2^ of nanofibre. Lanes 2–7, 0.1 cm^2^ of nanofibre per lane. VP60 levels were compared by fluorescent western blot using an anti- RHDV VP60 antibody and near IR fluorescent secondary antibody. Image acquisition and relative band intensities were obtained using a LiCor Odyssey FC instrument and Image Studio software. Figure S2: Gating strategy for T cell proliferation analysis; Figure S3: Gating strategy for the in vivo cytotoxicity.
