# Supplementary material for: Dry Formulation of Virus-Like Particles in Electrospun Nanofibers
Source: Vaccines (Basel). 2021 Mar 3;9(3):213. doi: 10.3390/vaccines9030213 (PMC8000389; doi:10.3390/vaccines9030213)
Supplement: Supplementary file 1 [file vaccines-09-00213-s001.zip › Suppl Fig 2.pdf]

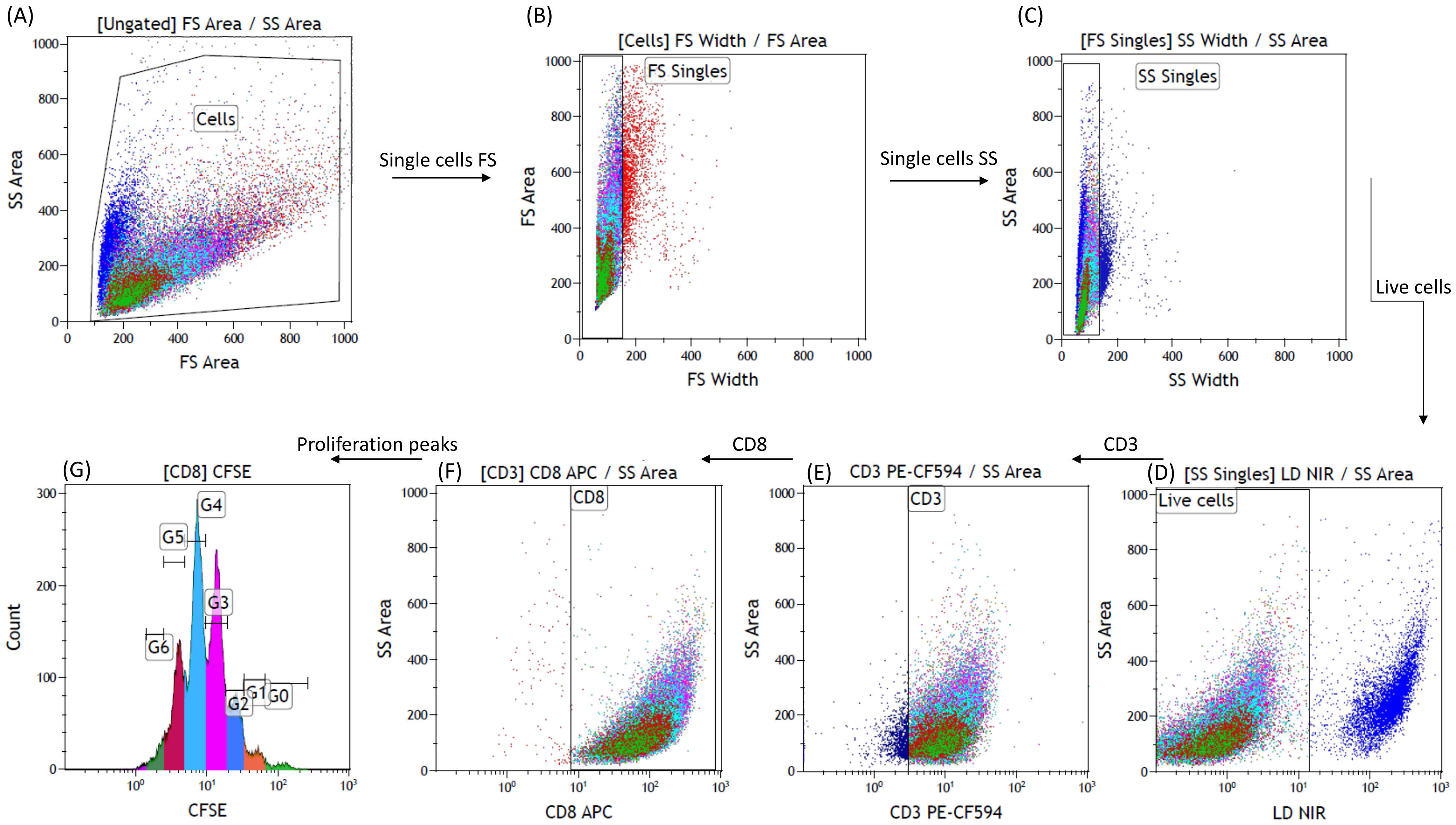

Supplementary Figure 2: Gating strategy for T cell proliferation analysis

Total cells (A) were first gated from debris by forward and side-scatter area, followed by the exclusion of doublet cells (B-C) by forward and side-scatter area against forward and side-scatter width. Dead cells were then excluded based on detection of the Near-IR live/dead stain (D). T cells were subsequently selected for by gating for CD3 positive cells stained with CD3-PECF594 (E). CD8 positive T cells were selected for by gating for cells stained with CD8 APC (F). Cells were finally gated into proliferation generations based on the amount of CFSE stain present (G)
