# Supplementary material for: Dry Formulation of Virus-Like Particles in Electrospun Nanofibers
Source: Vaccines (Basel). 2021 Mar 3;9(3):213. doi: 10.3390/vaccines9030213 (PMC8000389; doi:10.3390/vaccines9030213)
Supplement: Supplementary file 1 [file vaccines-09-00213-s001.zip › Suppl Fig 3.pdf]

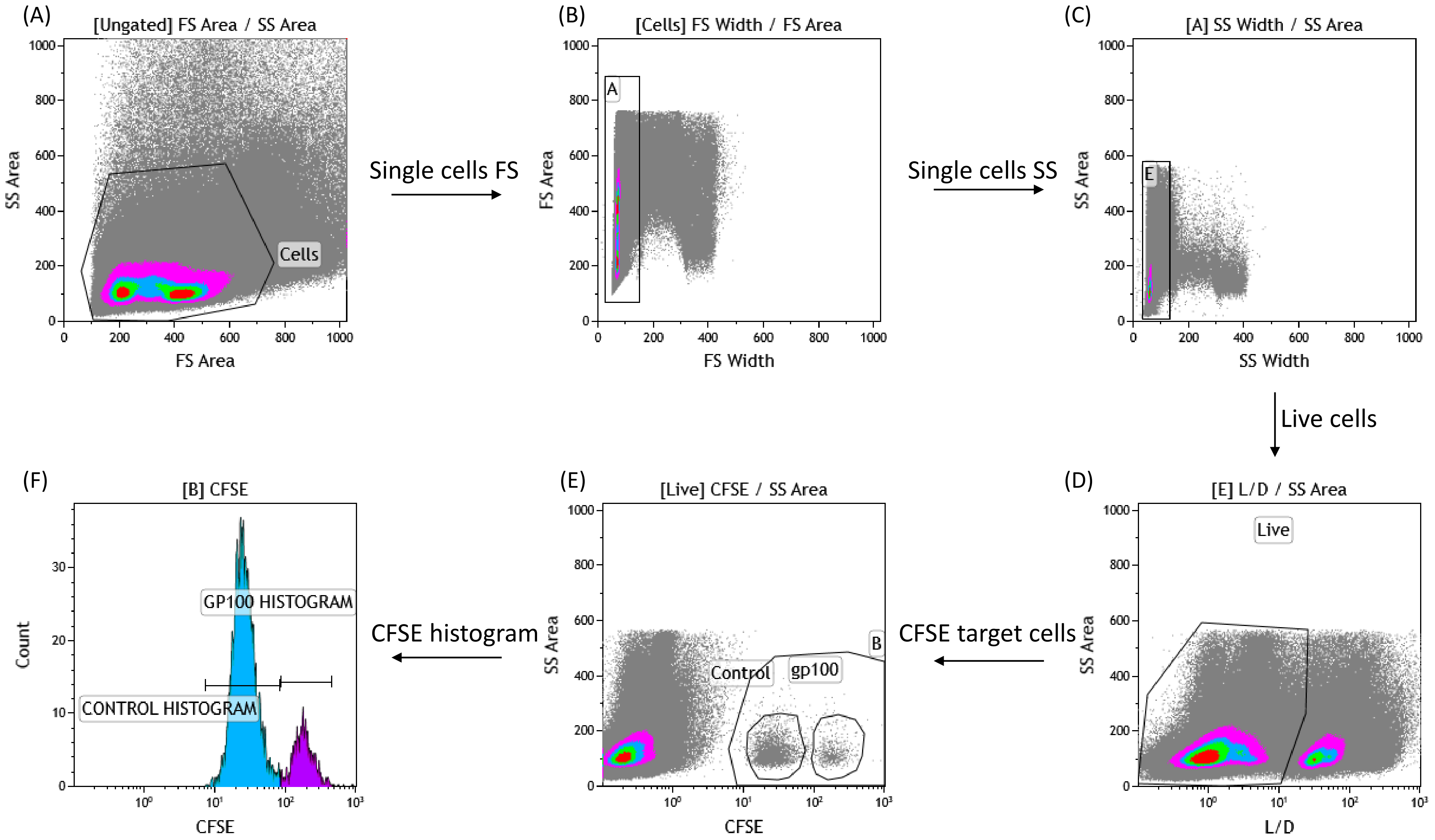

Gate	Number
All	3,740
CONTROL HISTOGRAM	3,095
GP100 HISTOGRAM	634

Gate	%Gated
Live	74.29

Supplementary Figure 3: Gating strategy for the *in vivo* cytotoxicity

Total cells were first gated from debris by (A) forward and side-scatter area, followed by the exclusion of doublet cells (B-C) by forward and side-scatter area against forward and side-scatter width. Dead cells were then excluded based on detection of the Near-IR live/dead stain (D). Control cells and gp100-pulsed cells were then separated based on their differential low and high staining with CFSE and counted (E-F).
