# Supplementary material for: Dry Formulation of Virus-Like Particles in Electrospun Nanofibers
Source: Vaccines (Basel). 2021 Mar 3;9(3):213. doi: 10.3390/vaccines9030213 (PMC8000389; doi:10.3390/vaccines9030213)
Supplement: Supplementary file 1 [file vaccines-09-00213-s001.zip › Suppl Fig 1.pdf]

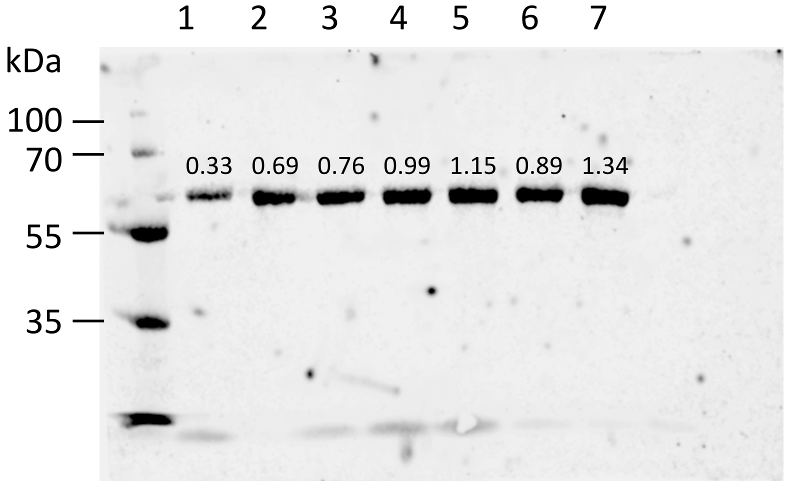

Supplementary Figure 1. Nanofibre loading uniformity. Lanes 1-7 represent randomly selected samples cut from the nanofibre mat. Lane 1, 0.05 cm^2 of nanofibre. Lanes 2-7, 0.1 cm^2 of nanofibre per lane. VP60 levels were compared by fluorescent western blot using an anti-RHDV VP60 antibody and near IR fluorescent secondary antibody. Image acquisition and relative band intensities were obtained using a LiCor Odyssey FC instrument and Image Studio software.
